# How adolescents understand their values: A qualitative study

**DOI:** 10.1177/1359104520964506

**Published:** 2020-10-19

**Authors:** Iona Lewis-Smith, Laura Pass, Shirley Reynolds

**Affiliations:** 1 School of Psychology and Clinical Language Sciences, University of Reading, Reading, UK; 2 Department of Clinical Psychology and Psychological Therapies, Norwich Medical School, University of East Anglia, UK

**Keywords:** Values, adolescents, development, qualitative, behavioural activation for the treatment of depression, acceptance and commitment therapy

## Abstract

An important component of some psychological therapies is the use of clients’ values to motivate behaviour change. Values are understood to be developed during childhood and adolescence but there has been limited exploration of how young people experience values and their function across contexts. This study aimed to explore adolescents’ understanding of the concept of ‘values’ and to elicit their experiences of values. Semi-structured, individual interviews were conducted with 11 adolescents aged 12–17 years. Thematic analysis was used to identify themes. Young people were readily able to discuss the meaning of ‘values’ and their own personal values. Three main themes were identified: (1) what values are (in general, and specific to themselves), (2) where values come from (relationships, education, growing up), and (3) why values are important (prioritising/decision making, reflecting on values is helpful). The adolescents in this study demonstrated an in-depth understanding of the meaning, origins and functions of values. The results suggest young people may welcome and benefit from opportunities to discuss their values, including in therapy.

*‘. . . you know your values, but it’s only when you talk about it that you understand where it comes from, how it’s changed’* [Millie, 17].

Values are often assumed to influence the development and expression of attitudes, beliefs, behaviour and wellbeing. Given the breath of their assumed role, research on human values has been conducted across a range of disciplines including sociology, psychology and anthropology. Perhaps unsurprisingly therefore, conceptual definitions of the term ‘values’ are varied (Rohan, 2000). Most psychological research on individuals’ values is based on Schwartz (1992), who suggested that values are ‘transsituational goals, varying in importance, that serve as guiding principles in the life of a person or other social entity’ (Schwartz, 1994, p. 21). Schwartz proposed that individuals’ values are derived from three fundamental needs: biological, social and group survival. He further argued that all human values sit within a circular structure and can be classified using four broad categories: conservation, self-enhancement, openness to change, and self-transcendence (Schwartz et al., 2012).

Values are thought to guide behaviours and remain relatively consistent during adulthood (Bardi & Schwartz, 2003; Vecchione et al., 2016), although they can change in response to events or experiences (e.g. Sortheix et al., 2019). There is evidence that values develop throughout childhood and adolescence and that the likelihood of value change decreases with age (Cieciuch et al., 2016; Daniel & Benish-Weisman, 2019). Young people often have similar values to their family (Boehnke et al., 2007) and value similarity between adolescent friends also suggests that peer relationships influence value development (Solomon & Knafo, 2007).

The Schwartz value structure can be observed in children as young as five (Lee et al., 2017). However, although childhood and adolescence appear to be critical periods in the development of values, there is very little research focussing on the consequences and significance of values in young peoples’ lives. Qualitative methods are well suited to exploration of the personal significance and experience of values but, similarly, there has been very limited qualitative research on values. Thus, a comprehensive understanding of values during adolescence and young people’s experiences of values across contexts is currently missing.

Despite the limited research on how values develop or are experienced, the concept of individual values is central to some psychotherapeutic approaches. For example, in Acceptance and Commitment Therapy (ACT: Hayes et al., 2011), Behavioural Activation for the Treatment of Depression (BATD: Lejuez et al., 2001, 2011, Narrative Therapy (Freedman & Combs, 1996) and Meaning Therapy (Wong, 2010) it is proposed that values are fundamental to psychological wellbeing and functioning.

Adolescence is an important period in the development of identity and autonomy (Fleming, 2005; Kroger et al., 2010) and a period when mental health problems often emerge for the first time (Kessler et al., 2005). Both ACT and BATD have been adapted for adolescents and these adaptations incorporate identifying adolescents’ values to form the basis of behavioural intervention (Hayes & Ciarrochi, 2015; Pass et al., 2015). However, although these psychological therapies assume that values are developmentally relevant and important to young people, no research has provided a direct understanding of how young people experience their values and how they understand their function across contexts. Thus, knowing how adolescents conceptualise their values would provide new insights beyond the existing quantitative explorations of values, as well as informing psychotherapeutic approaches that use individuals’ values to enhance psychological wellbeing. Therefore the aim of this study was to gain rich, detailed data on values in the lives of adolescents.

## Method

### Study design

One-to-one, semi-structured interviews were conducted with young people by the first author. Qualitative thematic analysis methodology (Braun & Clarke, 2006) was used to analyse the interview content. This study adopted a predominately linear-sequential approach to analysis (Kennedy & Thornburg, 2018) because most interviews were conducted before data analysis began; however, field notes from previous interviews were considered when conducting each subsequent interview. Data analysis was heavily data-driven, as opposed to theory-driven, given the paucity of existing qualitative and quantitative examinations of adolescents’ values.

### Participants and recruitment

Forty-one adolescents from year 8 and year 12 in a co-educational secondary school with sixth form in Berkshire, UK, were invited to take part in this study. They were contacted following their participation in a school survey conducted by the research team, where students provided consent to receive communications about future research studies. According to the school’s most recent Ofsted report, the majority of pupils at the school were White British and only a small number did not speak English as their first language. There were no exclusion criteria for the study and all participants who opted-in were interviewed. Participants were given an information sheet explaining the study and the need for them (or a parent) to give consent to the research. Parental consent was gained for those participants under the age of 16. Students aged 16 and above provided their own informed consent. All participants consented for their demographics and Short Mood and Feelings Questionnaire (SMFQ; Angold et al., 1995) responses collected in the school survey to be used in this study. Ethical approval for the study was obtained from the University of Reading’s School of Psychology and Clinical Language Science Research Ethics Committee.

Eight year 12 students (two males, six females, all White British, average age 16.75 years) and 3 year eight students (all female, all White British, average age 12.33 years) took part. Participant demographics and scores on the SMFQ are presented in Table 1. Pseudonyms have been used to protect participant confidentiality. Participants’ SMFQ scores ranged from 1 to 13 with a mean score of 6.5.

**Table 1.** Participant demographics and SMFQ scores.

Pseudonym	Gender	Age	SMFQ score
Charlotte	Female	12	8
Erin	Female	13	3
Isabelle	Female	16	6
Jessica	Female	12	1
Joshua	Male	17	4
Leo	Male	17	3
Millie	Female	17	13
Olivia	Female	17	6
Poppy	Female	16	11
Sophie	Female	17	6
Zara	Female	17	10

SMFQ = short mood and feelings questionnaire.

### Measures

The SMFQ assessed participants’ symptoms of depression, to contextualise the sample in relation to adolescents that psychological therapists may work with. The SMFQ consists of 13 items (e.g. ‘I felt miserable or unhappy’) that are scored as 0 = ‘Not true’, 1 = ‘Sometimes true’, and 2 = ‘True’. Scores on the SMFQ range from 0 to 26, with higher scores indicating more depression symptoms. In a sample of 11-17 year old primary care attendees, a SMFQ criterion score of 6 had a 80% sensitivity and 81% specificity to a diagnosis of depression (Katon et al., 2008) and in a community sample of 8-16 year olds, a criterion score of 8 had a sensitivity of 75% and a specificity of 74% (Thapar & McGuffin, 1998).

For demographic information, participants were asked to report their date of birth, gender, school year and ethnicity.

### Procedure

#### Interview procedure

Semi-structured interviews with participants were conducted using a topic guide to facilitate responses, while allowing the direction and content of each interview to be determined by each participant. The interview topic guide included questions that aimed to elicit participants’ understanding of values as a concept (e.g. *What does the word ‘values’ mean to you?*), what their values meant for them (e.g. *What do your values mean for your life?*) and how they thought about and expressed their values (e.g. *Tell me how you think about your values? How do you express your values?*).

All interviews were conducted at school during the school day. At the start of the interview, the interviewer explained the purpose of the interview, the terms of confidentiality, the format of the interview and expected length. All participants consented for their interview to be audio recorded and for the researcher to take brief notes during the interview. Interviews lasted between 20 and 60 minutes. One participant asked for the interviewer to define the term ‘values’, for which the interviewer used a description of values adapted from Brief Behavioural Activation (Brief BA; Pass et al., 2015) to form the basis of her answer, as this used language intended to be developmentally appropriate for adolescents. Apart from using the description of values from Brief BA for one participant, the first author tried to remain aware of and minimise the bias from her own understanding of values.

#### Data analysis procedure

Data analysis was undertaken following the thematic analysis procedures described by Braun and Clarke (2006). All interviews were transcribed verbatim by the first author using Nvivo 11 software, before repeated re-reading to familiarise her with the young peoples’ responses. Two cycles of line-by-line coding were used with the aim of interpreting and capturing the essence of the data (Saldaña, 2015). The first author then grouped the codes into semantically-related categories, determined by perceived patterns in the codes. Grouping of codes into potential themes was an iterative process, with the aim of refining categories and moving from concrete to more abstract categorisations (Saldaña, 2015). Next, the second and third authors coded a transcript blind to the themes identified by the first author and a discussion between the researchers was used to refine the themes. An iterative process of deciding appropriate labels for themes and their definition was then conducted by all three authors, after which the first author compared the final themes against the audio-recordings to check that all themes accurately reflected the content and meaning of the interviews. Throughout data analysis, the authors reflected on the influence of their assumptions and biases, including their understanding of values from the perspective of psychological therapies and their understanding of adolescent development.

## Results

### Overview of themes

**Figure 1.**
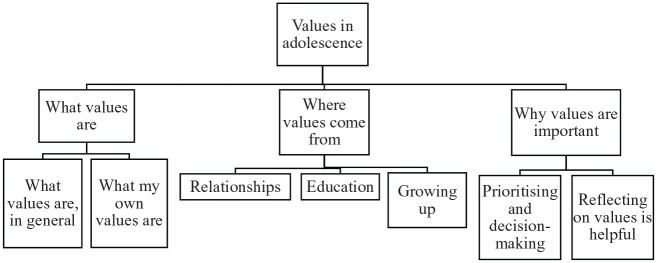
Thematic map showing the interview topic (level 1), themes (level 2) and subthemes (level 3).

Participants’ discussions of values were captured in three main themes (displayed in Figure 1): (1) what values are, (2) where values come from and (3) why values are important.

### Theme 1: ‘What values are’

This theme encompasses what the young people understood values to be as an abstract concept and also what they understood their personal values to be.

#### Subtheme: ‘What values are, in general’

All but one participant easily described what the term ‘values’ meant. Participants used a range of related concepts to explain what they understood values to be. These included ‘*morals*’, ‘*what you believe in*’, ‘*aspirations*’ and ‘*traits*’. Some participants also highlighted a link between values and the concept of the self, in other words, values *‘make you, kind of, the person you are’*. Most participants were able to view values as an abstract concept, for example, Joshua explained *‘[values are] less sort of physical things, more links you’ve made with people or various other aspects of life’*. The only participant who was unsure of what ‘values’ were, Zara, said she thought values were beliefs but she was not confident in this description. After asking the interviewer to explain their understanding of the term, she was reassured and continued to talk about them with ease.

#### Subtheme: ‘What my own values are’

Most participants spoke about their own values as being *‘good*’ or *‘right*’ and participants spontaneously described their values. These frequently included: relationships and the quality of relationships (e.g. ‘*my family’, ‘trust’*); education, learning and working hard (e.g. *‘I think important to me the most is probably my education’*); self-improvement and best self (e.g. *‘be the best I can be’*); enjoyment and happiness (e.g. *‘what’s the most important is just to be happy’*); independence (e.g. *‘I’d like to be able to just sort of go out in the world and sort of do my own thing’*); and responsibility (e.g. *‘being responsible’*).

### Theme 2: ‘Where values come from’

Young people talked fluently about how their values developed and what had influenced them.

#### Subtheme: ‘Relationships’

All participants stated that their values had been influenced by their family. Some participants said that adult family members had taught them their values through praise, punishment or by modelling value-congruent behaviour. For example, Isabelle said:

*‘my aunty works for a charity which helps children who have disabilities, or have lost relatives, I think. And they kind of, that’s what’s kind of helped guide me into thinking I wanna help someone make a difference’.*

The majority of participants also mentioned the influence of peers on value development. Participants described the bi-directional association between friendships and values – some friendships were formed because of shared values and some values were formed through friendships. Highlighting the causal link between friendships and value similarity, Leo said ‘*if I had a different friend group, yeah, I think my value of working hard could definitely have changed, 100 percent’.* On the other hand, Millie talked about how her friendship group were drawn to each other because of their shared values:

*‘I think that is kinda how friendship groups work, cos it’s like another friendship group who like who are quite like bitchy, talk about each other and stuff, like we wouldn’t really do that and they would. But like that’s why they’re a group of friends and we’re a group of friends. Like we might be friends with each other, but you do kind of gravitate towards the people that are gonna be similar to you’.*

Although most participants discussed how values developed through supportive peer relationships, Poppy described how being bullied by her peers had reinforced her value of ‘kindness towards others’:

*‘when I was younger maybe like being picked on a little bit probably makes you think like “I never want anyone else to go through what I went through”. . . just be like kind to everyone all the time so that no one ever has to like feel upset’.*

All participants recognised that, in addition to their own values being influenced by their relationships, other peoples’ values were dependent upon ‘*how they were brought up*’. Leo emphasised this point by saying ‘*our socialisation, I think, is key to everything*’. The extent to which participants accepted others’ individual differences, which they attributed to their different social environments, was varied. For example, Olivia reflected on how learning about her peers’ different backgrounds had informed her own values by making her ‘*realise how like lucky [she is]*’. However, she was also clear about her appreciation for peoples’ different values and priorities:

*‘I value like people’s personalities. And like I value other people’s values almost. I like how everyone has different things that they’re so like courageous about, and like I love how other people who are ambitious do different things, and how people find other things like so important that I sometimes don’t’.*

By contrast, some participants described how other peoples’ different values might cause them to behave in ways they considered *‘just not important at all’* or *‘really strange’* and that they ‘*would try [their] best to influence them’.* Despite this, most participants shared the sentiment that they would just have to accept peoples’ differences, even if this was sometimes difficult. Leo summarised this by saying: *‘I don’t agree with what you say but I’ll defend the right for you to say it’*. Participants therefore demonstrated that, alongside understanding how their own values developed, they critically evaluated where other peoples’ values came from and understood how this influenced the way they related to them.

#### Subtheme: ‘Education’

A few participants explained that they had been made aware of the concept of values at primary school, with Jessica reporting that ‘*in primary school we were really big on values*’. Participants also said they talked about and developed their values in certain school classes, including English, Politics, and Personal, Social, Health and Economic Education. Olivia highlighted the significance of school in personal development by saying that by going to school each day she ‘*might find something out that like completely alters the way that [she] think[s]*’. She reflected on how, when she was younger, she had ‘*less knowledge of the world*’ but as she has learned more about the world, she has ‘*become interested in like the world [she] live[s] in*’ and ‘*that’s where a lot of [her] values have come from*’. Leo talked a lot about the effect of education on his values. He said that he valued ‘*working hard*’ in order to ‘*achieve*’ at school and that ‘*the results at the end*’ of his hard work reinforced this value for him. Studying politics, in particular, seemed to have a big influence on Leo, as he explained it made him look at ‘*the bigger picture*’ and thus lead to him to feel that ‘*[knowing] how the world works is a big value*’. He reflected overall that ‘*I value school in a sense of its important in developing you as a person*’.

#### Subtheme: ‘Growing up’

Some participants talked about their value development as aided or enabled by the development of other cognitive or psychological processes. For example, Erin referred to how developments in inhibitory control altered peoples’ ability to behave in line with their values:

*‘if you wanted to be honest, except you really wanted that chocolate cake that you weren’t allowed to have, then you’d take it and you’d lie. Whereas, now I wouldn’t steal and then lie, I would rather just badger the other person for it until they said yes’.*

Three participants – Poppy, Zara and Millie – talked about how they thought their ability to realise and enact their values was enabled by increasing independence in thought and action as they got older. Poppy described how she had come to value standing up for what she believes in:

*‘when I was younger. . . I would never like really stand up. . . and like never really say anything if something was going on that I didn’t agree with and then I started thinking like “no, why am I doing that? Like it’s something that I believe in, if like something’s happening that I don’t want to be happening, then I should say something”. . . I think that’s like really important’.*

Millie echoed this idea when she described how, as she matured, she learned to follow her own values, as opposed to following others:

*‘as you grew up, you’re like “this isn’t right for me, it might be alright for you, but that’s ok, I’m not going to do it” kind of thing. And just like, yeah, definitely, I think just like maturing over the situations to be honest’.*

Other participants talked about learning to be less ‘*selfish*’ with age, as well as accruing knowledge from applying their values, learning from mistakes and seeing that ‘*values will change slightly after*’. Isabelle noted how becoming more conscious of the consequences of her behaviour with age made her aware of her values:

*‘when I was little, I just did things that I enjoyed. I never really took much notice. I kinda just did it but now I kind of realise that I do those things for a reason and I kind of think that other people do certain things for a reason’.*

Overall, participants acknowledged the importance of general developmental processes associated with growing up on the development, awareness and implementation of their values.

### Theme 3: ‘Why values are important’

Young people in this study had clear views about the importance of values to them personally and in general. Their views highlighted two distinct functions for their values.

#### Subtheme: ‘Prioritising and decision-making’

All participants talked about their values as being an important factor in making decisions and prioritising. Through knowing their values, participants said that they were able to decide what actions or outcomes were most important to them, both in the short-term and long-term. For example, Joshua was very clear about valuing his friendships above everything else, therefore he saw the importance of working hard to do well in his exams so he could go to university, as this would allow him to stay in close contact with his friends:

*‘I don’t want to end up redoing, um, my sixth form, because if I end up with bad grades at the end of it and have to redo it, but all my friends end up going off to uni, then it’s gonna be weaker connections with them, and maybe break quite a few of them’.*

Sophie also indicated that she was doing a ‘*lot of work towards*’ her future exams so she could ‘*get to where [she] want[s] to be*’ and that this was to train as an occupational therapist because she valued ‘*helping people*’.

Most participants mentioned the need to find a ‘*balance*’ between priorities and to decide the ‘*right value*’ to enact in a given situation. They also talked about situations where prioritising goals based on their values had been difficult and less rewarding in the short-term than simply emulating the behaviour of others, following their impulse, or satisfying their immediate desires. For example, Millie really valued learning and working hard, however, she talked about how it was sometimes difficult to follow these values:

*‘I should be revising but I wanna sit and watch TV. Like you know, you have to be, take responsibility for your education and stuff but you’re like “nah, I can’t really be bothered” kind of thing’.*

Despite acknowledging times when behaving in line with values could be difficult, a few participants also highlighted that their values provided hope and motivation, as opportunities for behavioural expression of their values allowed them to access a sense of fulfilment. Isabelle summarised this idea by saying:

*‘I would say that [values], kind of, like kind of gets you up in the morning, in a way. So, cos I kind of think it’s quite nice that you wake up and then you finish the day realising that you kind of helped someone, or you made someone feel confident or you expressed an emotion, or helped them express emotion as well. I kind of think that’s a good thing’.*

Zara - the only participant to express any uncertainty about what her values were - said that her uncertainty about what was most important to her made decision making difficult and reduced her motivation to invest effort in her schoolwork. She said:

*‘I think I need to do well [in my exams], but it’s just finding the motivation to actually revise, and to actually like do something about it. Like I think because my heads like quite up, like it’s all quite confused’.*

By highlighting the difficulties she experienced making decisions without clear values to guide her, Zara’s account further highlights how important values were in helping guide and motivate the young peoples’ behaviours.

Despite reflecting during the interviews on the role of values in directing their actions, all participants said that integrating their values in day-to-day decision-making was something they did automatically. Millie summarised this idea by saying, in relation to living according to her values, that:

*‘it is my decisions, like what is right and wrong and I’ve lived with that for, you know, like 17 years and it’s what I do without necessarily thinking about it’.*

#### Subtheme: ‘Reflecting on values is helpful’

Despite indicating that thinking about and behaving according to their values was an automatic process, at the end of the interview most participants also commented that having had the opportunity to consciously think and talk about their values had been a positive, helpful experience. For example, Millie said that talking about values could aid self-awareness and growth and, consequently, it should be encouraged:

*‘it is good to talk about it, because I feel like you get much more of an awareness about actually where you’ve come from and the journey that you’ve taken and, actually, like reflecting on yourself. So like you know, if you did find something, like you could think “oh, I need to change that” kind of thing. Like I think it is good, like I think it should be encouraged, kind of, more than it is’.*

Other participants said that, although values were something they’d ‘*never really talked about before*’, being ‘*open*’ and ‘*honest*’ about them during the interview felt ‘*good*’, ‘*nice*’ and Jessica said she ‘*quite enjoyed it*’. Over half of the participants also conveyed that values were ‘*personal*’ and therefore would not be something they would discuss with everyone but would be happy to talk about with some people ‘*depending on what the reasoning behind talking*’ about them was. For example, Charlotte said she’d be comfortable talking about her values with ‘*family and. . . my closest friend*’, a sentiment which Jessica echoed when she said of discussing her values: ‘*most friends I would be fine talking to*’.

## Discussion

This is the first study to explore how adolescents think, feel and talk about their values. This was achieved through thematic analysis of semi-structured interviews with young people at their school. The results demonstrate that almost all participants were readily able to access and communicate their values and that they saw their values as playing an instrumental role in their lives.

Abstract thinking and the self-concept further develop during adolescence (Dumontheil, 2014; Sebastian et al., 2008), thus it might be expected that adolescents would struggle to think and talk about values. However, all participants in this study (those 12–13 and 16–17 years old) could reflect on and communicate the content, meaning and purpose of their values. This suggests using the concept of values to support psychological therapies with young people is very likely to be achievable.

The young people had a strong sense of where their values came from and identified the main sources of influence as their relationships with family and friends. Participants described bidirectional associations between their values and friendships, whereby shared values drew them to build friendships, and their friendships influenced and reinforced their values. Giordano (2003) suggests that adolescents are attracted to others they perceive as similar to themselves and are more open to influence from those they want to befriend. The role of family influence was also acknowledged; despite the pervasive myth that young people reject the influence of parental relationships during adolescence, parents continue to play an instrumental role in providing support and guidance throughout adolescence (Smetana et al., 2006). Overall, participants’ perceptions of the origins of their values are consistent with findings from other research, which indicate that children often attribute some of their values to their parents (Whitbeck & Gecas, 1988) and that peoples’ values tend to be similar to those of their friends (Lea & Duck, 1982).

In addition to discussing the role of relationships in value development, the participants discussed maturing and the role of formal education. Nurturing the development of values is a part of school curricula in the UK and other countries (Lovat, 2011) and teachers consider the instruction and modelling of values to be an important aspect of their role (Thornberg, 2013). It is encouraging that, for the participants in this study, these efforts made an impact and it suggests that professionals working with adolescents can and do make a positive contribution to the development of young peoples’ values.

The significance of values in prioritising and decision-making was discussed. Participants saw their values as important in motivating behaviour and helping them to prioritise and make decisions. Studies have shown there are significant associations between values and reported behaviour (Bardi & Schwartz, 2003), and that priming self-transcendence values (such as benevolence) can increase prosocial behaviour (Arieli et al., 2014). It is significant that the participants in this study perceived an association between their values and behaviour, since this supports the theoretical propositions of BATD and ACT that values, motivation and behaviour can be causally and consciously linked.

Although the adolescents in this study indicated they did not often talk about their values, they also reported that discussing their values in the interview was a positive or worthwhile experience. This suggests adolescents may appreciate opportunities to articulate and develop their understanding of their values with people and in settings they feel comfortable with, which could include therapy.

While the purpose of this qualitative study was not to generalise to all adolescents, nevertheless it is a limitation that all participants were White British and the majority of were female, sixth form pupils from the same school. While the process of value content and structure development during adolescence does not substantially differ according to gender or ethnicity (Daniel & Benish-Weisman, 2019), adolescents’ experiences of value development may be associated with demographic factors. Furthermore, while some participants reported moderate levels of depression symptoms, therapists may find that adolescents experiencing Major Depressive Disorder or other mental health disorders might struggle with the cognitive demands of exploring their values in depth. The authors considered and reflected on their pre-existing ideas about adolescent values throughout the study, however it is acknowledged that the authors’ subjective perspectives will have necessarily influenced the research process. Further research is needed to address the potential cultural, ethnic, gender or age differences in adolescents’ experiences of values, as well as the impact of mental disorder on adolescents’ ability to access and articulate their values.
